# Clarification of Taxonomic Status within the *Pseudomonas syringae* Species Group Based on a Phylogenomic Analysis

**DOI:** 10.3389/fmicb.2017.02422

**Published:** 2017-12-07

**Authors:** Margarita Gomila, Antonio Busquets, Magdalena Mulet, Elena García-Valdés, Jorge Lalucat

**Affiliations:** ^1^Microbiology, Department of Biology, Universitat de les Illes Balears, Palma de Mallorca, Spain; ^2^Institut Mediterrani d'Estudis Avançats (Consejo Superior de Investigaciones Científicas—Universidad de las Islas Baleares), Palma de Mallorca, Spain

**Keywords:** *P. syringae*, phylogenetic group, phylogenomic species, core genome, pangenome, ANIb, GGDC, MLSA

## Abstract

The *Pseudomonas syringae* phylogenetic group comprises 15 recognized bacterial species and more than 60 pathovars. The classification and identification of strains is relevant for practical reasons but also for understanding the epidemiology and ecology of this group of plant pathogenic bacteria. Genome-based taxonomic analyses have been introduced recently to clarify the taxonomy of the whole genus. A set of 139 draft and complete genome sequences of strains belonging to all species of the *P. syringae* group available in public databases were analyzed, together with the genomes of closely related species used as outgroups. Comparative genomics based on the genome sequences of the species type strains in the group allowed the delineation of phylogenomic species and demonstrated that a high proportion of strains included in the study are misclassified. Furthermore, representatives of at least 7 putative novel species were detected. It was also confirmed that *P. ficuserectae, P. meliae*, and *P. savastanoi* are later synonyms of *P. amygdali* and that “*P. coronafaciens*” should be revived as a nomenspecies.

## Introduction

The genus *Pseudomonas* is divided into two phylogenetic lineages (*Pseudomonas aeruginosa* and *Pseudomonas fluorescens*) based on inferred evolutionary relationships by using multilocus sequence analysis (MLSA) of four housekeeping genes (Mulet et al., 2010). The *P. fluorescens* lineage contains six phylogenetic groups, one of them represented by *Pseudomonas syringae*, and includes most of the phytopathogens within the genus *Pseudomonas* (Bull et al., 2010).

*P. syringae* was described by Van Hall (1902) and several closely related species have since been described. In the Approved List of Bacterial Names (Skerman et al., 1980), three other species of phytopathogenic *Pseudomonas* were also included: *Pseudomonas cichorii* (Stapp, 1928), *Pseudomonas viridiflava* (Burkholder, 1939), *Pseudomonas caricapapayae* (Robbs, 1956), and *Pseudomonas amygdali* (Psallidas and Panagopoulos, 1975). “*Pseudomonas coronafaciens*” (Schaad and Cunfer, 1979) was not included in the Approved List of Bacterial Names and is not recognized as a valid species name. Until that moment, species characterizations and proposals have been performed using physiological, biochemical, serological, and pathological traits. Later, several other species closely related to *P. syringae* were proposed and validated: *Pseudomonas meliae* (Ogimi, 1977), *Pseudomonas savastanoi* (Gardan et al., 1992), *Pseudomonas ficuserectae* (Goto, 1983), *Pseudomonas avellanae* (Janse et al., 1996), *Pseudomonas cannabina* (Gardan et al., 1999), *Pseudomonas tremae* (Gardan et al., 1999), *Pseudomonas congelans* (Behrendt et al., 2003), *Pseudomonas asturiensis* (González et al., 2013), *Pseudomonas cerasi* (Kałuzna et al., 2016), and *Pseudomonas caspiana* (Busquets et al., 2017). The *P. syringae* species complex is usually considered to include all these taxonomically closely related species.

Molecular techniques based on experimental DNA-DNA hybridizations (DDH) or on DNA sequence analysis are essential in determining actual taxonomy. DDH were used first in the *P. syringae* group of species by Pecnold and Grogan (1973) and when *P. savastanoi* was proposed (Gardan et al., 1992). Gardan and colleagues established eight genomic groups, called genomospecies, based on DDH analysis (Gardan et al., 1999) that allowed the reclassification of strains previously known as pathovars of *P. syringae* as the new species *P. cannabina* and *P. tremae*. A phylogenetic study based on the 16S rRNA gene sequences of species in the genus was applied first by Moore et al. (1996) to propose a phylogenetic scheme within the genus, but until the description of *P. avellanae*, sequence analyses were not included in new species proposals in the *P. syringae* species complex. Due to the limitations in sequence variation in the 16S rRNA gene, other genes have been used for species delineation, especially the *rpoD* gene (Yamamoto et al., 2000; Mulet et al., 2010; Parkinson et al., 2011) and the *cts* gene (Berge et al., 2014). These analyses have allowed the delineation of phylogenetic groups, or phylogroups, within the species complex. Multilocus sequence analyses (MLSA) based on the sequences of three or four housekeeping genes have also been very successful in clarifying the phylogeny of strains in the *Pseudomonas* genus (Mulet et al., 2010; Bull et al., 2011; Berge et al., 2014). More specifically, Almeida et al. (2010) have developed the Plant associated microbes database (PAMDB) that contains sequences for MLST and MLSA accessible in a useful website. As a result of the molecular techniques, many strains have been reclassified and a more stable phylogenetic classification has become possible. Determining the precise taxonomic affiliations of strains in the *P. syringae* species complex can be difficult when pathovars are considered (Baltrus, 2016; Vinatzer et al., 2017). Currently, the species in the *P. syringae* phylogenetic group are subdivided into over 60 pathovars defined by pathogenic characters, 15 genomospecies are defined by DDH, 13 phylogroups are defined by MLSA using 3 or 4 genes, and 15 validly described species are accepted in the List of Prokaryotic Names with Standing in Nomenclature (Parte, 2014; http://www.bacterio.net/). Vinatzer and Bull (2009) have published a comprehensive history of the taxonomy of plant pathogenic bacteria, the use of MLSA and the impact of genomic approaches on taxonomy of plant pathogenic bacteria.

Genome-based taxonomic analyses have been recently introduced, and several algorithms are currently used for strain comparisons, such as the average nucleotide identity based on BLAST or MUMmer algorithms (ANIb, ANIm) and genome-to-genome distance calculations (GGDC), and are substituted for experimental DDH (Konstantinidis and Tiedje, 2007). Comparative genomics provides another tool that allows core genome and pangenome analyses at different levels of classification in a phylogenomic approach, that is, phylogenetic inference by combining many genes (Jeffroy et al., 2006). Recently, it has been proposed the use of similarity-based codes, called life identification numbers (LINs) to name individual bacterial isolates in the *P*. *syringae* species complex (Vinatzer et al., 2017).

As noted by Morris et al. (2017), “delineation of pertinent phylogenetic contours of plant pathogenic bacteria and naming of strains independent of their presumed life style is one of the five challenges for understanding the ecology of plant pathogenic bacteria.” With the goal of clarifying the taxonomic delineation of species in the *P. syringae* phylogenetic group, 139 genomes of the 15 recognized species assigned to this group that are available in public databases have been analyzed by a phylogenomic approach. At least one member of each phylogroup described in the *P. syringae* phylogenetic branch by Berge et al. (2014) was included in the analyses if it was available in the public databases. “*P. coronafaciens*” strains and the three closely related species in the *Pseudomonas lutea* group (*P. graminis, P. lutea*, and *P. abietaniphila*) were also included, as well as an unclassified *Pseudomonas* sp. strain S25 isolated in our laboratory. MLSA and several *in silico* algorithms for genome comparisons (e.g., ANIb, GGDC) allowed the clustering of strains in 6 clear genomic branches. Core genome and pangenome analyses have been performed in the present study for the whole *P. syringae* phylogenetic group, for 5 of the 6 individual genomic branches and for 7 proposed phylogenomic species to explore their usefulness to delineate inter- and intra-species relationships. We included in our study the genome sequences of the type strains in the *P*. *syringae* group and 19 of the 56 pathotype strains recently published by Thakur et al. (2016) with the main purpose to clarify the species delineation, without considering all the pathovars. Species affiliation of the pathotypes is a prerequisite for the posterior study of the phylogeny of the pathovars.

## Materials and methods

### Data collection and genome sequences

Draft and complete genome sequences of 139 strains belonging to different species of the *P. syringae* group available in the NCBI database were analyzed, including the genomes of 3 *P. aeruginosa* strains and 2 *P. stutzeri* strains used as outgroup. The 139 selected strains included the genomes of 15 species type strains of the *P. syringae* phylogenetic group, *Pseudomonas* sp. S25 and representatives of “*P. coronafaciens*.” Genomes of strains in the *P. lutea* phylogenetic group (*P. lutea, P. graminis*, and *P. abietaniphila*) were used as an outgroup in the analysis because they belong to the closest phylogenetic group to the *P. syringae* group (Gomila et al., 2015). Six type strain genomes were analyzed in duplicate: five were representatives of the same type strain but from two different culture collections, and the sequence of *P. cannabina* was deposited twice by 2 different authors. The set of 139 genome sequences of *Pseudomonas* was retrieved from the GenBank database on 30th April 2017. The list of the 139 complete or draft genomes analyzed and additional details are provided in Supplemental Table S1.

### Three-gene multilocus sequence analysis (3-gene MLSA)

An MLSA based on the analysis of the partial sequences of the 16S rRNA, *gyrB* and *rpoD* genes was performed. The sequences of the 16S rRNA, *gyrB*, and *rpoD* genes were extracted from each genome studied and compared with the corresponding sequences of all *Pseudomonas* species type strains (161) described through 2016. Sequences are available in the public National Center for Biotechnology Information (NCBI) database. A concatenated gene tree was constructed using the individual alignments in the following order: 16S rRNA (1,309 nt), *gyrB* (803 nt), and *rpoD* (791 nt) by methods previously described (Gomila et al., 2015). Genomes that did not contain the 16S rRNA, *gyrB*, and *rpoD* gene sequences were removed from the MLSA concatenated analysis.

### Whole-genome comparisons

*In silico* tools were used for genomic species delineation. Average nucleotide identity based on BLAST algorithm (ANIb) was calculated between all pairs of genomes, using the JSpecies software tool available at the webpage http://www.imedea.uib.es/jspecies (Konstantinidis and Tiedje, 2007; Richter and Rosselló-Móra, 2009). The recommended species cut-off was 95%. The similarity matrix obtained with all pairwise genomic comparisons was used to generate a UPGMA dendrogram using the PAST software. GGDC was performed between genome pairs on specific sets of genomes using the GGDC 2.0 update available in the web service http://ggdc.dsmz.de (Meier-Kolthoff et al., 2013).

### Phylogenomic comparisons

#### Pangenome analysis and clustering

A comparative genomic analysis was performed using the GET_HOMOLOGUES software described by Contreras-Moreira and Vinuesa (2013). All genomes were annotated with PROKKA for comparison purposes (Seemann, 2014), and the protein amino acid sequences obtained were compared using the criterion of 50% similarity over 50% of coverage alignment. Core genome and pangenome analyses were performed with three different clustering algorithms, bi-directional best-hits (BDBH), COGtriangle (COG), and OrthoMCL (OMCL). The four clusters determined from the analyses were defined as previously described (Koonin and Wolf, 2008; Kaas et al., 2012): core, soft core, shell, and cloud. Core genome and pangenome analyses were performed for all the genomes analyzed and for subsets of them.

#### Phylogenomic analysis (core MLSA)

All proteins codified by genes of the core genome that were present in monocopy were aligned, and the resulting alignments were concatenated. Elimination of poorly aligned positions and divergent regions of protein sequences were performed with Gblocks (Castresana, 2000), and the phylogenetic tree was constructed with the PhyML program (Guindon et al., 2010). Analysis of the concatenated amino acid sequences of the core proteins (core MLSA) was performed for all genomes and for the different delineated subsets.

#### Average amino acid identities among homologous CDSs

A GET_HOMOLOGUES script was used to estimate the average amino acid identities of CDSs between individual members of specific pangenome clusters. Gower's distance matrix were determined based on the percent amino acid identities of protein coding genes in the different genome branches using a script from GET_HOMOLOGUES Those distance matrices obtained were further illustrated as heatmaps, showing similarities and differences between genomes.

## Results

### Genome characteristics

Genome characteristics of the strains studied are summarized in Supplemental Table S1. The genome sequences of the 15 species type strains so far described in the *P. syringae* phylogenetic group were included. At least one member of 11 of the 13 phylogroups described in the *P. syringae* phylogenetic branch by Berge et al. (2014) was included in the analyses. Genomes of phylogroups 8 and 12 were not available in public databases. As a control, the sequences of the genomes of the *P. cichorii, P. viridiflava, P. congelans*, and *P. meliae* type strains were studied in duplicate, i.e., two type strains from two different culture collections. Two genome sequences of *P. cannabina* ICMP 2823^T^ with two different accession numbers were also studied. The studied genomes included 121 genomes with a status of “contig” (the number of contigs ranged from 5 to 5,099; mean: 617 contigs) and 6 with a status of “complete genome.” The chromosome sizes ranged from 4,713,747 to 7,317,256 bp (mean: 5,976,989 bp) and the GC content in mol % ranged from 56.95 to 59.38 (mean: 58.34). Plasmids were reported in the databases for only 3 of the 6 closed genomes: *P. savastanoi* pv. *savastanoi* strain 1448A (2 plasmids, 3% of the genome content), *P. cerasi* strain 58^T^ (6 plasmids, 7% of the genome), and *P. syringae* pv. *tomato* DC3000 (2 plasmids, 2% of the genome). The plasmids were not included in the comparative analyses.

The authenticity of the type strains studied was checked by analyzing their affiliation in the *Pseudomonas* 3-gene MLSA tree (Gomila et al., 2015). All type strains were affiliated with the previously determined gene sequences with the exception of 2 species type strains, *P*. *tremae* and *P. lutea*. The genome of *P. tremae* ICMP 9151^T^ clustered close to those of “*P. coronafaciens”* strains, and the sequences were different from those published for *P. tremae* LMG 22121^T^. Therefore, the published sequences of the *cts, gyrB, rpoB, rpoD*, aconitase, and 16S rRNA genes of the type strains of three different culture collections (LMG 22121^T^, CFBP 3229^T^, NCPPB 3465^T^) were compared with the corresponding sequences of the *P. tremae* ICMP 9151^T^ genome. The sequences were only 88–98% identical. We concluded that the status of the species type strain of *P. tremae* ICMP 9151^T^ must be revised, and therefore it was not further considered as a type strain in the present study. Two genome sequences are available for *P. lutea* type strains. Surprisingly, *P. lutea* LMG 21974^T^ was an outlier. The 16S rDNA gene sequence of strain LMG 21974^T^ was 99% identical to that of *Pseudomonas poae* DSM 14936^T^ in the *P. fluorescens* phylogenetic group. Several housekeeping genes of strain LMG 21974^T^ were analyzed, and it was concluded that the deposited *P. lutea* LMG 21974^T^ genome did not belong to the *P. lutea* phylogenetic group. The rest of the duplicated genome sequences were concordant.

### Phylogenomic analysis with outgroups

All 139 genomes were phylogenetically analyzed using different strategies: (i) 3-gene MLSA of the partial sequences of the 16S rRNA, *gyrB* and *rpoD* genes, (ii) a phylogenomic tree based on the concatenation of all single-copy conserved protein sequences that conforms the core genome of the 139 genomes analyzed (core MLSA), and (iii) by ANIb.

**Figure 1:**
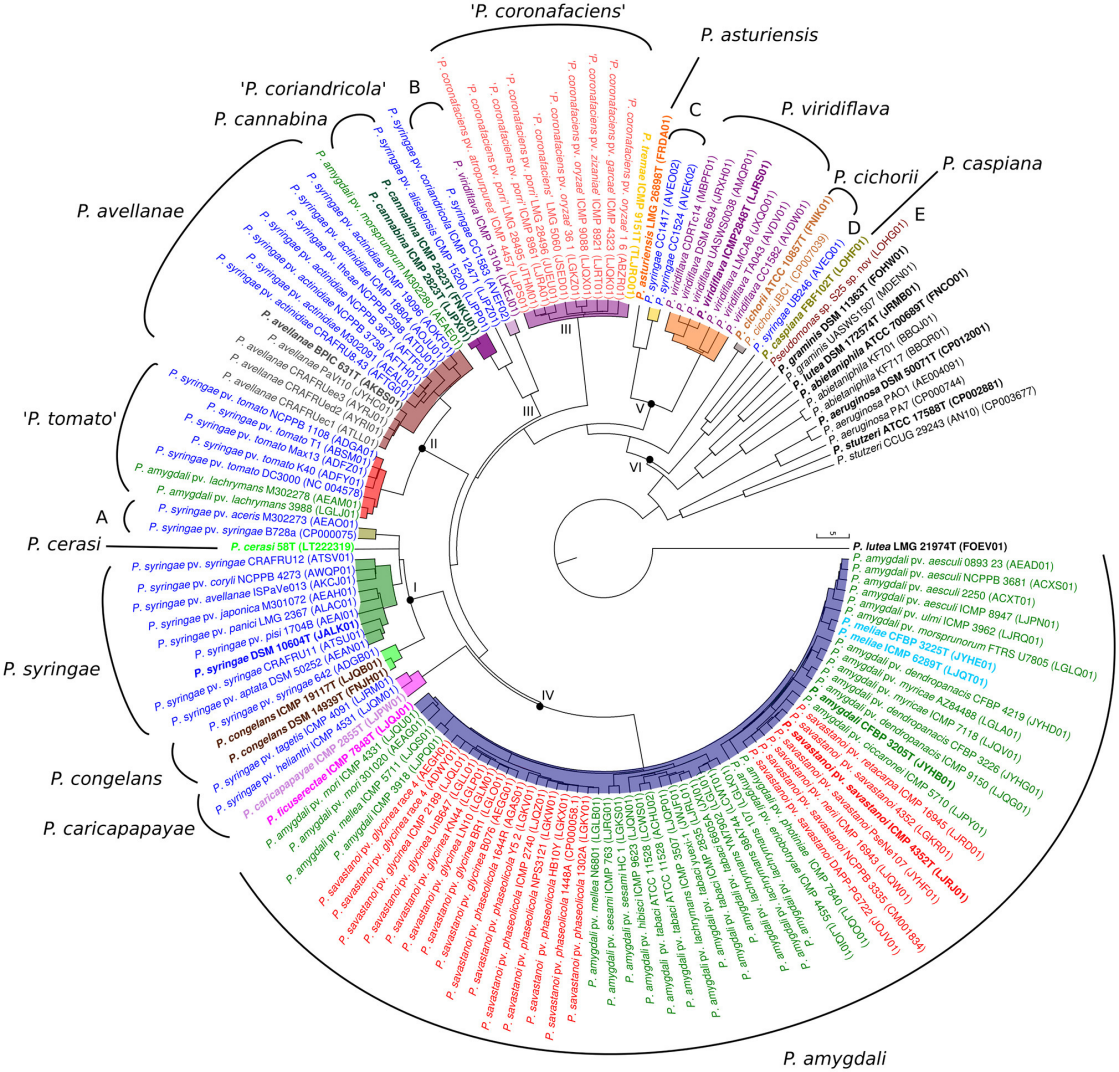
UPGMA dendrogram based on ANIb values of all pairwise comparisons. Each species name, as submitted in the database, is labeled with different colors. Roman numerals at the corresponding nodes indicate phylogenomic branches defined. Phylogenomic species inside each phylogenetic branch are highlighted with different colors. Species type strains are labeled in bold. Accession numbers of the corresponding genomes are given in brackets. Proposed phylogenomic species are indicated in the external circle. Putative novel species are marked in quotation marks or by capital letters (A–E).

(i) The 3-gene MLSA phylogenetic analysis included the 15 species type strains in the *P. syringae* phylogenetic group that are validly described and were used in a previous publication (Gomila et al., 2015) combined with the 139 *Pseudomonas* complete or draft genomes available in databases. A phylogenetic tree (Supplemental Figure S1) was generated based on the concatenated sequences with a total length of 2,796 nucleotides. One hundred and nine of the 139 strains (78% of the genomes analyzed) were affiliated with the corresponding species type strain, and their species assignments were considered correct.

(ii) One hundred and forty-nine monocopy genes were defined in the core genome of the whole set of 139 genomes. The phylogenomic tree obtained after the concatenation of the amino acid sequences of the 149 monocopy genes (core MLSA) is shown in Supplemental Figure S2. The *P. aeruginosa* and *P. stutzeri* genomes were used as outgroups. From the 33,744 positions obtained after the concatenation of the individual alignments, 93% of them were finally analyzed (31,400 positions). Bootstrap values were indicated on the nodes. In this phylogenomic tree, six main clusters or phylogenomic branches could be detected, indicated in Roman numerals from I to VI (Supplemental Figure S2).

(iii) Average nucleotide identities based on BLAST (ANIb) were calculated for the 139 genomes, obtaining a square matrix with 19,321 pairwise comparison values (Supplemental Table S2). A dendrogram was generated for this matrix to assess phylogenetic coherence (Figure 1). The ANIb dendrogram showed high topological congruence compared with the 3-gene MLSA phylogenetic tree and with the core MLSA phylogenetic tree. All duplicated type strains clustered together with ANIb values higher than 99.87%. The reference genomes of *P. aeruginosa, P. stutzeri*, and the species in the *P. lutea* group clustered outside the *P. syringae* group. All ANIb percentage values calculated were plotted on a graph that demonstrated a clear gap between 89 and 93% (Supplemental Figure S3). Only 206 values were observed between 93 and 96%, <2%, and corresponded to pairwise comparisons among strains in groups I and II. Six genomic branches were again delineated in the *P. syringae* group based on the observed tree branching in the ANIb dendrogram with an ANIb cut-off of 93%, such that members of different genomic branches could not have an ANIb of >93%. The genomic branches corresponded to the same six main phylogenetic branches detected in the 3-gene MLSA and in the core-gene phylogenetic tree. Therefore, each branch was considered a homogeneous phylogenomic branch, without taxonomic implications, to later facilitate comparative genomic analyses. The only exception detected was genomic branch II, which was divided into two clusters by ANIb value. The boundary values of the phylogenomic branches (minimal value among strains of different phylogenomic branches) were higher than 4.8%. The usually accepted cut-off for species delineation based on the ANIb lies between 95 and 96% (Richter and Rosselló-Móra, 2009). In branch I (represented by *P. syringae*) and branch IV (represented by *P. amygdali*), no clear gap at the 95–96% ANIb cut-off could be delineated; therefore, we were not able to distinguish genomic species (genospecies or genomospecies) by using only the ANIb value. Groupings of strains with intrabranch values higher than 94.3% in the other four branches were separated by clear gaps, and each group was considered a phylogenomic species. Seventeen clusters with ANIb intrabranch values higher than 94.3% could be differentiated, and their boundary values were higher than 4.4% with the exception of 9 strains in genomic branch I, which included the *P. syringae* type strain, as indicated in Table 1 and Supplemental Table S2. Each of the 17 clusters was considered a phylogenomic species. Genomic branches I, IV, V, and VI contain more than one validly described species.

**Table 1:** ANIb, GGDC, 3 genes-MLSA, and core MLSA indices for the delineation of the proposed phylogenomic species in each genomic branch.

**Genomic Branch**	**Phylogenomic species and representative strains**	**Nr of strains**	**ANIb**		**GGDC**		**3 genes–MLSA**		**Core MLSA**	
			**Minimal intra-cluster ANIb (%)**	**Closest inter-cluster ANIb (%)**	**Minimal intra-cluster GGDC (%)**	**Closest inter-cluster GGDC (%)**	**Minimal intra-cluster MLSA (%)**	**Closest inter-cluster MLSA (%)**	**Minimal intra-cluster Core (%)**	**Closest inter-cluster Core (%)**
I	*P. congelans*	3	98.42	94.15	87.7	58.7	99.64	98.64	99.7	98.7
	*P. syringae*	9	94.79	95.15	68.4	62.8	98.75	98.39	98.8	98.9
	*P. cerasi*	1	–	95.07	–	61.2	–	97.47	–	99
	Species A (strain B728a)	2	98.27	95.21	87.6	62.8	99.25	98.96	99.6	98.9
II	*P. avellanae*	13	96.66	95.41	73.2	65	99.03	98.85	99.2	99.1
	“*P. tomato*” (DC3000)	7	98.41	95.41	89	65	99.35	98.85	99.7	99.1
III	*P.cannabina*	3	97.49	95.17	70.9	62.8	98.89	98.42	99.5	98.9
	“*P. coriandricola”* (ICMP 12471)	1	–	95.17	–	62.8	–	98.42	–	99
	species B (strain CC1583)	2	98.91	89.53	90.9	38.8	99.71	96.81	99.7	97.5
	“*P. coronafaciens”* (LMG 5060)	11	97.68	86.45	74	32.9	99.28	96.84	99.7	95.9
IV	*P. amygdali, P. meliae, P. savastanoi, P. ficuserectae*	57	96.79	89.2	76.4	39	97.45	97.54	98.8	98.1
	*P. caricapapayae*	3	97.3	89.38	78.8	39	98.97	97.49	99.5	98.1
V	*P. asturiensis*	1	100	94.63	–	59	–	98.57	–	98.8
	species C (strain CC1417)	2	99.05	94.63	92.7	59	99.64	98.57	99.9	98.8
	*P.viridiflava*	7	96.39	86.69	71.6	32.8	98.24	95.2	99.6	96.8
VI	*P. cichorii*	2	99.94	81.35	100	23.6	100	92.26	100	91.3
	species D (strain UB246)	1	–	88.52	–	36.8	–	97.31	–	97.6
	*P. caspiana*	1	–	88.52	–	32.6	–	96.31	–	97.5
	species E (strain S25)	1	–	86.93	–	23.5	–	97.31	–	97.5

*The representative strain for the unnamed phylogenomic species is indicated in brackets*.

The results obtained for the three methodologies applied were compared. Strain clustering was maintained in the 3-gene MLSA at a cut-off of 97% (Table 1), which is in agreement with previous studies (Gomila et al., 2015), but some slight differences can be observed in the branching order (Supplemental Figure S1). Two strains with a 3-gene MLSA value lower than 97% cannot be assigned to the same species. Phylogenetic similarities in the analysis of the three concatenated genes were compared with the ANIb similarities calculated in the whole genome analysis and the results plotted in Supplemental Figure S4. A good correspondence between the ANIb and 3-gene MLSA indices could be observed. The six main clusters or genomic branches observed in the ANIb results were also detected in the core-gene MLSA tree, although phylogenomic branch III was divided into two closely related branches in the ANIb analysis.

GGDC similarities were also calculated for all genomes included in each ANIb/core MLSA genomic branch in order to clarify phylogenetic assignments to species. The results are shown in Supplemental Table S3 and were highly concordant with the ANIb values, accepting a species cut-off value of 70% as recommended by Meier-Kolthoff et al. (2013). It is worth mentioning that the 2 genomes available for the *P. cannabina* type strain were only 70.9% similar in the GGDC analysis but were almost identical in the ANIb (99.9% similar) and MLSA analyses (100% identical). This discrepancy has to be attributed to the *in silico* methodologies or to the quality of the genome sequences.

The combined use of the 4 indices allowed the delineation of 19 phylogenomic species, and these are described below in the context of each phylogenomic branch. ANIb values among members of different phylogenomic species were lower than 96%. Genomic branch I (intrabranch values: 93.11–98.18% for ANIb and 97.18–99.93% for 3-gene MLSA) included 15 strains divided into 4 groups, 3 of them belonging to recognized taxonomically described species: the *P. syringae* type strain and 8 closely related strains, *P. cerasi* (1 strain), and *P. congelans* (2 strains; the type strain is duplicated). The fourth group, designated as unnamed group A and represented by strain B728a, includes 2 strains. The boundaries at 95% ANIb were diffuse within this branch, although the 4 clusters could be clearly distinguished with GGDC values lower than the accepted cut-off of 70% (intrabranch values between the 4 groups ranged between 54.9 and 62.8%).

Genomic branch II, represented by the *P. avellanae* type strain and 19 other strains, presented intrabranch values of 94.30–99.99% for ANIb, 98.21–100% for 3-gene MLSA, and 59–99% for GGDC. Two homogeneous and clear sub-branches could be distinguished at a cut-off lower than 96% in ANIb (94.30–95.41%), which corresponded to 95.09% in 3-gene MLSA and values lower of 64% in GGDC and can be considered phylogenomic species. Seven strains, represented by *P. syringae* pv. *tomato* DC3000, conformed a possible phylogenomic species. They grouped with similarities higher than 98.41%. The other phylogenomic species, represented by the *P. avellanae* type strain, together with 12 additional strains, grouped at 96.6% of ANIb, and in both cases, GGDC values were higher than 70%. Each phylogenomic species was circumscribed by uniform intrabranch values.

Intrabranch values of genomic branch III were 85.15–99.9% ANIb, 93.90–100% 3-gene MLSA, and 30–99.9% GGDC. This genomic branch included 17 strains distributed in 4 possible phylogenomic species: one included 2 *P. cannabina* type strain genome sequences and another strain identified as *P. syringae*; *P. syringae* pv. coriandricola ICMP 12471 was a singleton; 11 strains (10 of them classified as “*P. coronafaciens*,” not taxonomically validly described, and the supposed type strain of *P. tremae*); and an unnamed phylogenomic species B (2 strains). The 4 sub-branches were also clearly distinguished at a threshold of 62% in GGDC.

Intrabranch values of genomic branch IV were 88.56–99.99% for ANIb, 96.13–100% for 3-gene MLSA, and 37.3–99.8% for GGDC. It included 5 validly described species type strains: the *P. meliae, P. amygdali, P. savastanoi*, and *P. ficuserectae* type strains, grouped together with 53 additional strains forming a unique phylogenomic species with clear boundaries from the rest of the strains; and *P. caricapapayae* (together with two additional strains). The 2 proposed phylogenomic species were clearly separated at the established cut-offs of 95% ANIb, 97% MLSA, and 70% GGDC.

Genomic branch V included two validly described species type strains with intrabranch values of 80.1–99.97% for ANIb, 91.68–100% for 3-gene MLSA, and 32.6–92.7% for GGDC. *P. asturiensis* was a singleton, and *P. viridiflava* was represented by 7 strains. Two strains formed a separate sub-branch (possible phylogenomic species C) close to *P. asturiensis*. The phylogenomic species were separated at cut-offs of 95% ANIb, 97% MLSA, and 70% GGDC.

Genomic branch VI was more distant and diverse. It included the type strains of *P. cichorii* and *P. caspiana*, together with other 2 strains: *Pseudomonas* sp. S25 and *P. syringae* UB246. The 4 strains were clearly separated at the accepted species cut-offs for ANIb, GGDC and 3-gene MLSA and have to be considered four different species.

As discussed later, these results suggested that a high proportion of genomes (53 of 127, 42%) were submitted in the databases with a species name affiliation different from that suggested by their affiliation in the ANIb, GGDC, 3-gene MLSA, and core MLSA dendrograms.

### Species assignations of pathovars

Sixty-two pathovars of *P. syringae* are listed in the “Comprehensive list of names of plant pathogenic bacteria, 1980–2007” (Bull et al., 2010). Twenty-seven strains of *P. syringae* assigned to 15 different pathovars were included in the present study (3 pathovars with more than 1 representative: pv. *actinidiae* 6 strains, pv. *tomato* 5 strains, and pv. *syringae* 4 strains) to emphasize that the correct species affiliation is a prerequisite for the posterior study of the phylogeny of the pathovars. The 6 pv. *actinidiae* strains clustered together with *P*. *avellanae* strains. The 5 strains of pv. *tomato* clustered also together in one phylogenomic species included in genomic branch II. On the contrary, the 4 strains of pv. *syringae* affiliated to three different phylogenomic species (*P. congelans, P. syringae*, and the proposed new phylogenomic species A) in branch I. The rest of pathovars represented by single strains were distributed in branches I, II, III, and IV. Only 8 of the pathovars were affiliated with *P. syringae* phylogenomic species (e.g., *P. syringae* pv. *tagetis* ICMP 4091 and *P. syringae* pv. *helianthi* ICMP 4531 belonged to *P. caricapapayae*). It is worthy of note that five of the six *P. syringae* pathotype strains included in this study did not affiliate with *P. syringae* genomic species: *P. syringae pv. tagetis* ICMP 4091 belongs to *P. caricapapayae*; *P. syringae pv. actinidiae* NCPPB 3739 belongs to *P. avellanae*; *P. syringae pv. alisalensis* ICMP 15200 belongs to *P. cannabin*a; and finally *P. syringae pv. coriandricola* ICMP 12471 was a putative new phylogenomic species. This suggested the need to reclassify the misclassified strains.

Most of the 14 pathovars of the 33 *P. amygdali* strains clustered in the same phylogenomic species with the exception of *P. amygdali* pv. *morsprunorum*, that clustered with *P. avellanae* strains, and 2 *P. amygdali* pv. *lachrymans* that clustered with *P. syringae* pv. *tomato* strains. The 5 pathovars of the 23 *P. savastanoi* strains clustered together in the same phylogenomic species represented by *P. amygdali*. The 5 pathovars of the 9 strains of *P. coronafaciens* affiliated to the same phylogenomic species. Only 3 pathovars (*lachrymans, morsprunorum*, and *syringae*) were assigned to more than 1 phylogenomic species.

### Core genome and pangenome analysis of the *P. syringae* group

To facilitate later comparative genomic analyses and due to the good correspondence between the 4 methods, the 6 genomic branches were maintained for further comparative genomic analysis (Figure 1, Supplemental Figure S2 and Table 1). Similarities between the members of different branches were always lower than 90% in ANIb, lower than 97% in 3-gene MLSA and lower than 70% in GGDC analyses.

**Figure 2:**
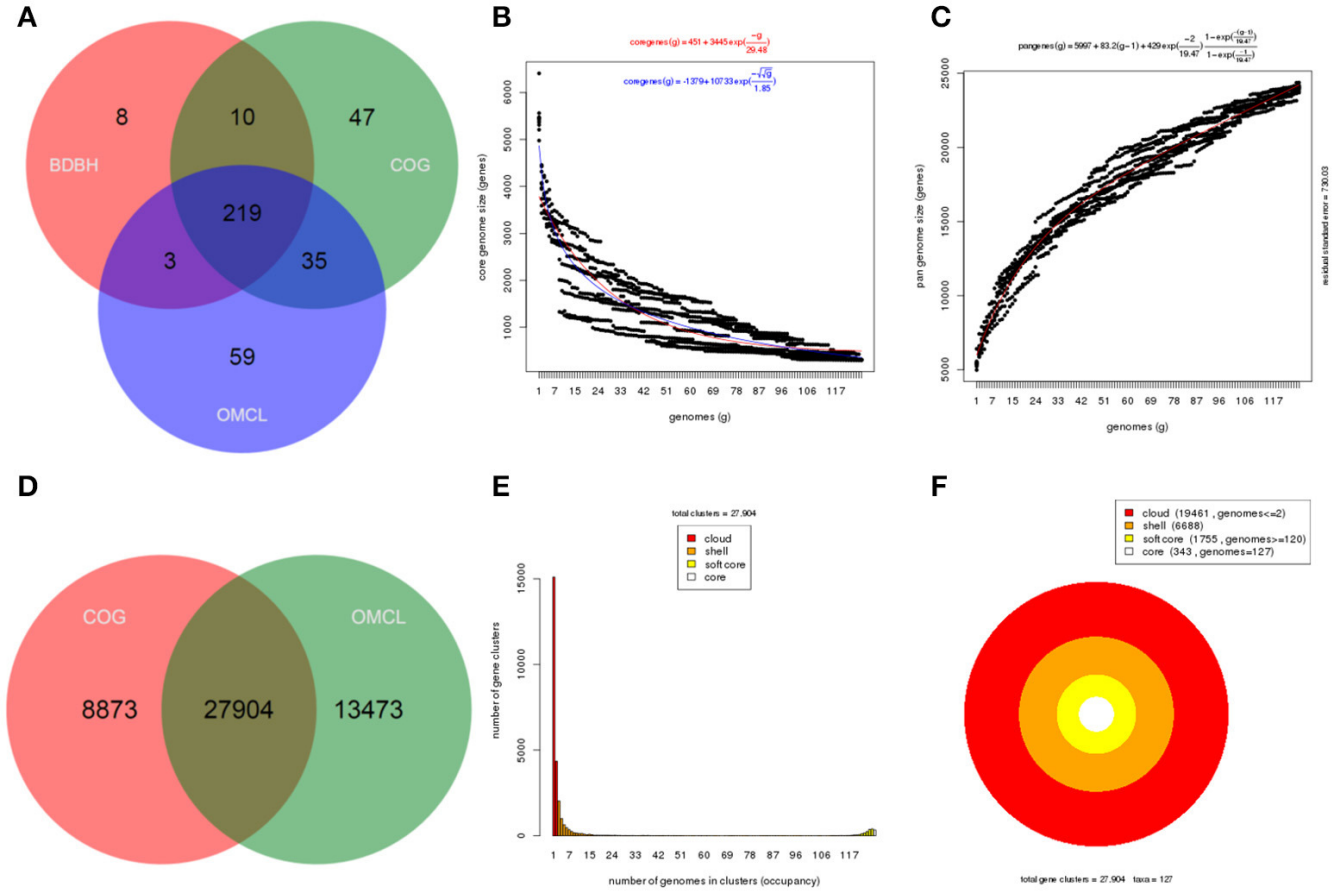
Core and pangenome analysis of the 127 strains in the *P. syringae* phylogenetic group. **(A)** Venn diagram of core genomes generated by the BDBH, COG, and OMCL strategies. **(B)** Estimate of core genome size with the Tettelin (blue) and Willenbrock (red) fits. **(C)** Estimate of pangenome size with the Tettelin fit. **(D)** Venn analysis of pangenomes generated by COG and OMCL. **(E,F)** Partition of the OMCL pangenomic matrix into shell, cloud, soft-core, and core compartments. These plots can be easily created with GET_HOMOLOGUES auxiliary scripts, as explained in the manual.

Core genome and pangenome analyses were performed for the whole set of strains selected belonging to the *P. syringae* group (127 genomes) and for each of the five genomic branches (I–V) delineated by the previous methodologies. Members of group VI were not included in the analyses because they were more distantly related and only 5 strains representing 4 phylogenomic species were available. Each set of genomes was analyzed with the GET_HOMOLOGUES software. Different images were produced for each pangenome analysis: (1) a Venn diagram of core genomes generated by the three algorithms BDBH, COG, and OMCL and of pangenomes generated by COG and OMCL algorithm, (2) the core genome size was estimated with the Tettelin and Willenbrock fits and the pangenome size with the Tettelin fit, and (3) the partition of the OMCL pangenomic matrix into shell, cloud, soft core, and core compartments (Figure 2, Supplemental Figures S5–S9).

**Figure 3:**
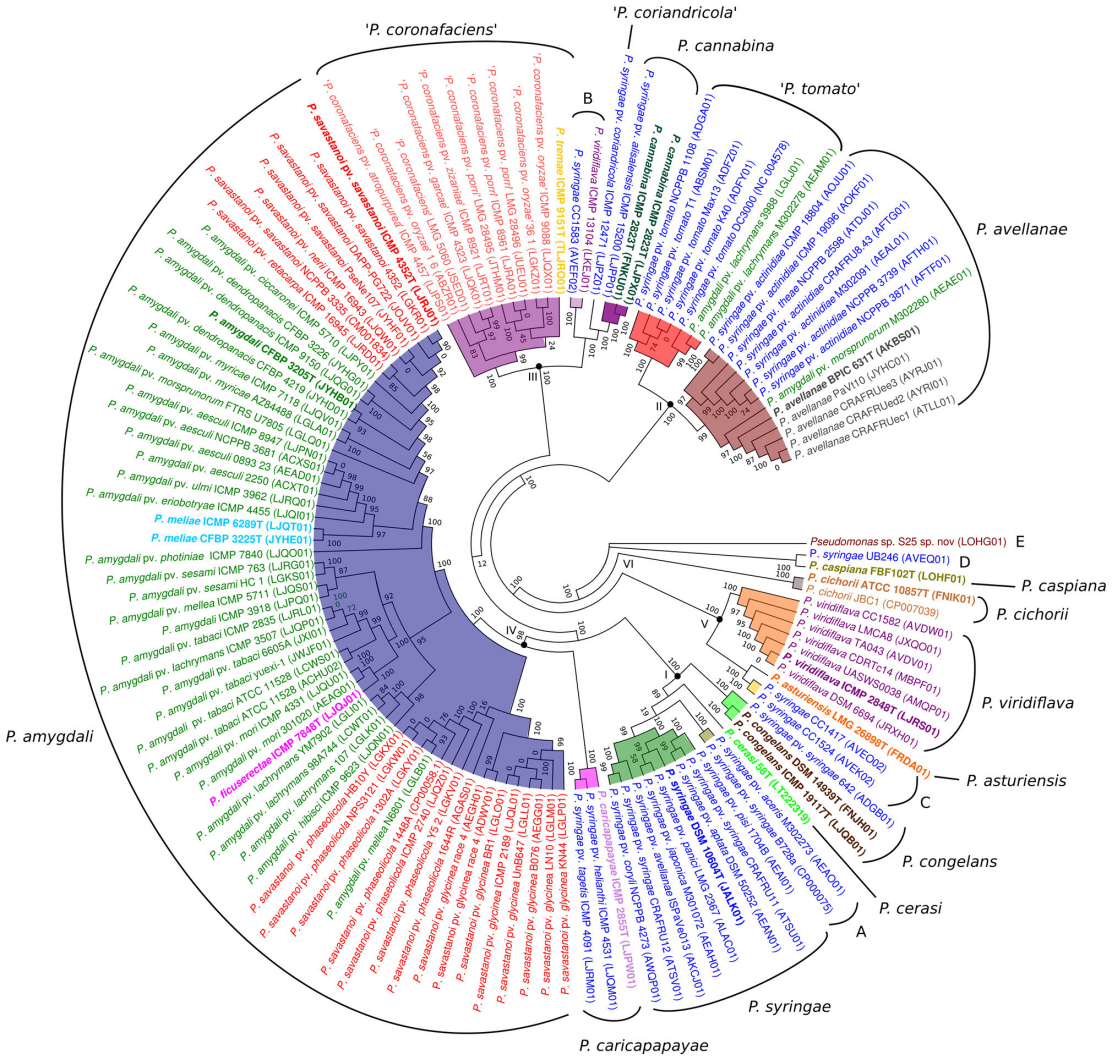
Phylogenetic tree of the concatenated amino acid sequences of 219 monocopy proteins of the core genome defined in the 127 genomes analyzed. Forty-three thousand one hundred thirty-three amino acid positions were used to construct the tree. Each species name, as submitted in the database, is labeled with different colors. Roman numerals at the corresponding nodes indicate phylogenomic branches defined. Phylogenomic species inside each phylogenetic branch are highlighted with different colors. Species type strains are labeled in bold. Accession numbers of the corresponding genomes are given in brackets. Proposed phylogenomic species are indicated in the external circle. Putative novel species are marked in quotation marks or by capital letters (A–E). All bootstrap values are indicated in the nodes.

The core genome of the 127 strains in the *P. syringae* phylogenetic group contained 343 genes. Two hundred and nineteen of them were in monocopy and were concatenated and analyzed to establish their phylogenetic relationships as previously described. The clustering of strains in the 6 phylogenetic groups was identical to those obtained with the other indices (ANIb, GGDC, 3-gene MLSA, and core MLSA of 139 genomes), showing the same branching order and supported by high bootstrap values (100) (Figure 3). Genomic branch II was the only exception, being separated from groups I, III, and IV with a bootstrap value of only 12. Bull et al. (2011) also observed this result. The 5 main phylogenomic branches were also supported by a high number of shared genes, representing the core genome proteins as a minimum of 20% of the whole genome, as indicated in Table 2.

**Table 2:** Core and pangenome analyses of the 127 strains included in the *P. syringae* phylogenetic group, as well as for 5 of the individual groups defined (I–V).

	***P. syringae* phylogenetic group**	**Group I**	**Group II**	**Group III**	**Group IV**	**Group V**
Nr of strains	127	15	20	17	57	10
Coregenome proteins	343	1,938	1,229	2,367	1,694	3,900
Pangenome proteins	27,904	13,150	13,493	11,873	16,810	7,704
Cloud	19,461	8,139	7,559	6,366	9,826	2,329
Shell	6,688	1,888	3,715	2,189	4,014	1,254
Soft core	1,755	3,123	2,219	3,318	2,970	4,121
% Conserved genes	6.29	23.75	16.45	27.95	17.67	53.49
% Flexible genome	93.71	76.25	83.55	72.05	82.33	46.51

*Numbers of genes in the shell, cloud, soft-core, and core compartments are indicated. Percentages of conserved genes and flexible genome are also given*.

Each branch was analyzed separately to assess the potential use of the shared genes for species delineation. Table 2 summarizes the number of core genome and pangenome genes calculated for all genomes and for each specific group analyzed. Table 2 shows the genes present in the “soft core” (genes present in 95% of the genome analyzed), in the “shell” cluster (genes moderately common in the pangenome, present in >10% and <89% of the genomes and in the “cloud” cluster (genes present in very few of the genomes analyzed, 2 or less). The “core” and “soft core” clusters include highly conserved genes with phylogenetic information (Bezuidt et al., 2016). The shell and the cloud clusters represent subsets of the flexible genome, which reflect the adaptation of strains to particular environments and also the evolutionary history these organisms. The 127 genomes of the *P. syringae* phylogenetic group show a high percentage of genes in the flexible genome, indicating that these strains are able to share genes and are highly diverse.

Average amino acid identity matrices were calculated using protein-CDSs within the 5 phylogenomic branches (Supplemental Figures S5–S9). The heatmap shows the clustering of genomes into different groups based on average similarities and differences of their CDS amino acid identities. In this case, core and flexible gene pools are combined. The clustering of the genomes in each phylogenomic branch follows the same groupings observed with the other methodologies (3-gene MLSA, core MLSA, ANIb, and GGDC). That is, in phylogenomic branch I, the four phylogenomic species detected with the previous methodologies could also be differentiated by clear boundaries.

The amino acid sequences of the core genes for each phylogenomic branch were concatenated, and the phylogenetic tree was constructed with PhyML. The phylogenetic trees are depicted in Supplemental Figures S5–S9. The clustering observed was consistent with the results obtained previously and supported the proposed phylogenomic species.

The core genome and pangenome were also analyzed for 6 delineated phylogenomic species that contained at least 7 strains each. Four of them included the type strains of the validly described species *P. syringae, P. avellanae, P. amygdali*, and *P. viridiflava*. Additionally, genomes of the proposed phylogenomic species “*P. coronafaciens*” and “*P. tomato*,” were also studied. The results are shown in Table 3 and Supplemental Figures S10–S15. The pool of conserved genes decreased with increasing genome number and represents at least 17% of the individual genomes. The percentage of the core genes in the 6 proposed phylogenomic species ranged between 17.67 and 72.60%. The low percentage of conserved genes might be directly associated with the difficulty to phylogenetically assign some strains to the correct phylogenomic species.

**Table 3:** Core and pangenome analyses of the phylogenomic species proposed with more than 6 strains in the *P. syringae* phylogenetic group.

	**Group I**	**Group II**		**Group III**	**Group IV**	**Group V**
	***P. syringae***	***“P. tomato”***	***P. avellanae***	***“P. coronafaciens”***	***P. amygdali***	***P. viridiflava***
Nr of strains	9	7	13	11	57	7
Coregenome proteins	2,185	4,197	1,329	2,888	1,694	4,438
Pangenome proteins	11,966	7,022	12,361	9,674	16,810	6,452
Cloud	7,210	1,605	7,007	4,512	9,826	1,244
Shell	1,394	875	2,952	1,145	4,014	524
Soft core	3,392	4,542	2,402	4,017	2,970	4,684
% Conserved genes	28.35	64.68	19.43	41.52	17.67	72.60
% Flexible genome	71.90	35.32	80.57	58.48	82.33	27.40

*Numbers of genes in the shell, cloud, soft-core, and core compartments are indicated. Percentages of conserved genes and flexible genome are also given*.

This low percentage of conserved genes correlates with a higher number of cloud genes that contribute to the flexible genome.

## Discussion

Although the taxonomy of *Pseudomonas*, and more specifically of the *P. syringae* phylogenetic group, has been extensively analyzed, significant uncertainties remain regarding the genus boundaries and species composition of this heterogeneous taxon. Many species have been named without adequate descriptions, or their identifications have not been updated with more modern techniques. Phylogenomic insights published by Gomila et al. (2015) and more recently by Tran et al. (2017) have substantially improved the knowledge of the whole genus. Vinatzer et al. (2017) used genome similarities to study the taxonomy of plant-pathogenic bacteria and constructed core genome phylogenies for plant-pathogenic bacteria. The composition of distinct species and whether *P. syringae* is a cohesive unit has been debated for a long time (Janse et al., 1996; Bull et al., 2010). Several recent publications have tried to clarify this situation (Baltrus, 2016; Baltrus et al., 2017), also at the pathovar classification level (Thakur et al., 2016).

Strains were originally identified phenotypically as members of the *P. syringae* complex if they were fluorescent pseudomonads, positive for levan sucrase activity, negative for oxidase activity, unable to rot potato, able to produce arginine dihydrolase and able to cause a hypersensitive response on tobacco (the LOPAT group 1 strains; Lelliott et al., 1966; Sands et al., 1970). In 1975, numerous formerly distinct LOPAT group 1 plant pathogenic species were combined into the species *P. syringae* (Lapage et al., 1975), and the confusion increased due to subspecific pathovar names have been given to distinct pathogenic characters and host of isolation (Young, 2008). At that time a large number of nomenspecies of these bacteria were defined and became widely regarded as host-adapted pathogenic varieties (pathovars). Consequently, the Approved Lists of Bacterial Names did not list most of these nomenspecies, which thus lost standing in nomenclature. Main reason were the absence of deposited strains in culture collections, lack of adequate phenotypic descriptions and phenotypic traits that distinguished the proposed species names. The International Society of Plant Pathologists published a checklist of the earlier nomenspecies and pathovars (Dye et al., 1980) and advised that such names should be revived only for the original bacteria (Lapage et al., 1992).

Classification based only on phenotype has led to increased taxonomic confusion, as more *P. syringae* strains have been isolated from different environments, including non-diseased tissues and environmental sources, such as rivers, lakes, snowfields, and clouds (Morris et al., 2008). Phenotypic diversity of strains in relevant species has also been demonstrated (Demba Diallo et al., 2012; Bartoli et al., 2014) and many strains cannot be easily classified. Currently, the *P. syringae* species complex is subdivided into over 60 pathovars defined by pathogenic characters, nine genomospecies defined by DDH and 13 phylogenetic groups (phylogroups) defined by multilocus sequence analysis (Sarkar and Guttman, 2004; Hwang et al., 2005; Almeida et al., 2010; Bull et al., 2011; Berge et al., 2014).

The aim of this work is to try to circumscribe the *P. syringae* species complex and classify its strains into species according to the taxonomic rules and thresholds actually accepted in taxonomy. Considering all methods tested together, we were able to circumscribe 6 phylogenomic branches within the *P. syringae* phylogenetic group.

The first branch, branch I, included 15 strains divided into 4 groups corresponding to four different phylogenomic species: *P. syringae, P. congelans, P. cerasi*, and novel species A. The first one includes the *P. syringae* type strain, together with only 9 strains previously identified as *P. syringae*. The rest of the *P. syringae* strains included in the present study (23) must be reassigned to other species (see Supplemental Table S1).

A second branch, branch II, contains 20 strains: 5 *P. avellanae* strains, including the species type strain, together with 1 strain classified as *P. amygdali* pv. *morsprunorum* (M302280), 5 strains of *P. syringae* pv. *tomato*, 2 of *P. amygdali* pv. *lachrymans*, 7 of *P. syringae* pv. *actinidiae*, and 1 of *P. syringae* pv. *theae*. We have included in this study five strains not considered in the previous publication by Scortichini et al. (2013) on *P. avellanae* genomes. The 3-gene MLSA values for all strains of this group ranged from 99.46 to 100% and divided these 20 strains into two subclusters. These 2 subclusters are maintained in the analysis of 219 concatenated core genes. All strains in the branch have ANIb similarity values ranging from 94.3 to 100%, but 2 sub-branches can be delineated: one at 97.1–100%, which corresponds to the group of the *P. avellanae* type strain, and another at 98.4–100%, which corresponds to strains of *P. syringae* pv. *tomato* and *P. amygdali* pv. *lachrymans*. These two sub-branches can also be delineated with the GGDC values; they are in accordance with Gardan's genomospecies 3 and 8, respectively, and with the phylogroups established by Parkinson and Berge. If we apply the currently accepted species threshold, all the strains of the group might be assigned to the species *P. avellanae*, but attending to all indices tested and the boundaries of the two subclusters, the possibility to differentiate two species or 2 subspecies must be considered. We have distinguished 2 distinct phylogenomic species: one is *P. avellanae*, and we propose the provisional operative name of “*Pseudomonas tomato*” for the branch that includes strain DC3000, pending a deeper taxonomic analysis. To formally propose a new species clear phenotypic characteristics that differentiate the new species with its closely related species have to be found. In our experience, the whole-cell protein profiles obtained by MALDI-TOF mass spectrometry can be a method of choice to phenotypically discriminate new species in the genus *Pseudomonas* (Mulet et al., 2012).

Branch III includes 4 phylogenomic species: 3 strains in one cluster must be assigned to *P. cannabina*; 1 strain deposited as *P. viridiflava* and another as *P. syringae* must be considered representatives of new species B; *P. syringae* pv. *coriandricola* ICMP 13104 represents a phylogenomic species provisionally named “*Pseudomonas coriandricola*”; 10 strains initially assigned to “*P. coronafaciens”* cluster together in all analyses and constitute the fourth phylogenomic species. This group includes the genome of *P. tremae* ICMP 9151^T^, not considered type strain in the present study. Therefore, the genome of *P. tremae* LMG 22121^T^ was sequenced and ANIb and GGDC results demonstrated that it has to be included in genomic branch IV (results not shown), that strengthen the possible misclassification of strain ICMP 9151^T^. The “*P. coronafaciens”* strains belong to phylogroup 4 of Parkinson and Berge and to genomospecies 4 of Gardan. The species “*P. coronafaciens*” has been proposed by Schaad and Cunfer (1979) based on phenotypic characteristics, and the indices studied clearly support the revival of “*P. coronafaciens”* as a nomenspecies.

Two phylogenomic species can be delineated in branch IV, which correspond to phylogroup 6 of Parkinson and Berge and genomospecies 7 of Gardan. Two strains and the *P. caricapapayae* type strain must be assigned to this species. The other phylogenomic species is more abundant and very homogeneous and contains 4 accepted nomenspecies. As already noted by Gardan et al. (1999), strains in this phylogenomic species have been assigned to genomospecies 3 and must be considered *P. amygdali* strains. The other 3 are later synonyms (*P. ficuserectae, P. meliae*, and *P. savastanoi*). As mentioned before *P. tremae* should be also considered in this group as a later synonym once will be assessed the correct genome.

Three well-defined phylogenomic species were distinguished in branch V. One was formed by *P. asturiensis* strains, another for a novel species C with 2 strains, and the other by *P. viridiflava* strains. Strains in this branch shared at least 53% of the genes in the pangenome. The strains *P. syringae* CC1417 and *P. syringae* CC1524 are considered non-phytopathogens, and *P. asturiensis* LMG 26898^T^ is phytopathogenic. The three strains are closely related in all the indices tested and were isolated from distant geographical areas (Montana, USA; France and Spain, respectively) and ecological habitats (rocks in waterfall in pristine woods, stream water and soybeans, respectively; Morris et al., 2008; González et al., 2013; Baltrus et al., 2014). The ANIb and GGDC indices are near the borderline of the species acceptance cut-off and share at least 87% of the pangenome in the Gower analysis. Consequently, strains CC1417 and CC1524 are not members of the *P. syringae* phylogenomic species and might be considered strains of *P. asturiensis*. However, the differences in plant pathogenicity and other practical reasons suggest that the taxonomic status of both strains merit further analyses before a definitive classification and must be considered for the moment representatives of putative new species C.

In branch VI, four phylogenomic species have been defined: the *P. caspiana* type strain is a representative of the other 4 strains of the species (Beiki et al., 2016; Busquets et al., 2017); *P. cichorii* with two strains, *Pseudomonas* sp. S25 and *P. syringae* UB246 are singletons, and more closely related strains are needed for a definitive taxonomic assignment of both these strains, which are assigned to putative new species D and E.

Overall, we were able to distinguish 19 phylogenomic species in the *P. syringae* phylogenetic group distributed within 6 phylogenomic branches. Two strains are assigned to 2 different phylogenomic species when the following criteria are accomplished: (i) ANIb value is lower than 94.5%, (ii) GGDC values lower than 68%, and (iii) 3-gene MLSA similarity lower than 98%. ANIb values between 94.5 and 96% might be analyzed carefully with respect to other characteristics such as GGDC, the core genome and pangenome analyses. In general, very good agreement has been found between these phylogenomic species, the phylogroups of Parkinson and Berge, and the genomospecies of Gardan. In fact, Bull et al. (2011) showed also how MLSA quite accurately reflects the genomospecies described by Gardan et al. (1999) by experimental DNA-DNA hybridizations.

A strain was assigned to a given phylogenomic species when it fell into one of the 19 phylogenomic species delineated as described above. In 58% of strains, there was an agreement between strain name and genomospecies. Furthermore, 23 out of 32 (72%) strains deposited as *P. syringae* were not assigned to the *P. syringae* phylogenomic species but were scattered among 10 different phylogenomic species. This fact points out the importance of correctly assigning a genome to the right species. Thanks to NGS technologies, a remarkable increase in the number of sequenced genomes, both draft and complete, are available, but the correct assignment of the sequenced strains to the corresponding species with the accepted taxonomic tools is important before comparative analyses with other genomes can be performed.

Genomic data are very useful in the actual taxonomy to delineate phylogenomic species that merits the species status. However, it is possible that many species will be separated in several species, even when the abundance of species names can be confusing. In this sense, the use by the experts in phytopathology will consolidate or not the use of these new species names. In many practical issues it can be maintained the less precise concept of *P. syringae* species complex for all of them, although it is essential a proper naming of bacterial species in order to establish a truly systematic taxonomy and avoid confusions in the scientific communities.

## Conclusions

Comparative genomics is a very useful tool for the establishment of a stable taxonomy, and we demonstrate its usefulness for the plant pathogenic bacteria studied in the present manuscript. Although further taxonomic studies are needed to support formal proposals, based on the present study of strains in the *P. syringae* phylogenetic group, we suggest that *P. ficuserectae, P. meliae*, and *P. savastanoi* are later synonyms of *P. amygdali* and, therefore, the group includes 11 recognized nomenspecies: *P. amygdali, P. asturiensis, P. avellanae, P. cannabina, P. caricapapayae, P. caspiana, P. cerasi, P. cichorii, P. congelans, P. syringae*, and *P. viridiflava*. Additionally, “*P. coronafaciens”* should be revived as a nomenspecies, and 27 strains representing 7 putative new species must be considered.

## Author contributors

MG, EG-V, and JL: conceived and designed the research project, and analyzed the data; AB and MM performed the experiments; MG: performed the bioinformatic analyses of the data; MG, AB, MM, EG-V, and JL: interpreted the results and contributed to the writing of the manuscript.

### Conflict of interest statement

The authors declare that the research was conducted in the absence of any commercial or financial relationships that could be construed as a potential conflict of interest.
