# Supplementary material for: Clarification of Taxonomic Status within the Pseudomonas syringae Species Group Based on a Phylogenomic Analysis
Source: Front Microbiol. 2017 Dec 7;8:2422. doi: 10.3389/fmicb.2017.02422 (PMC5725466; doi:10.3389/fmicb.2017.02422)
Supplement: Supplementary file 1 [file Image1.PDF]

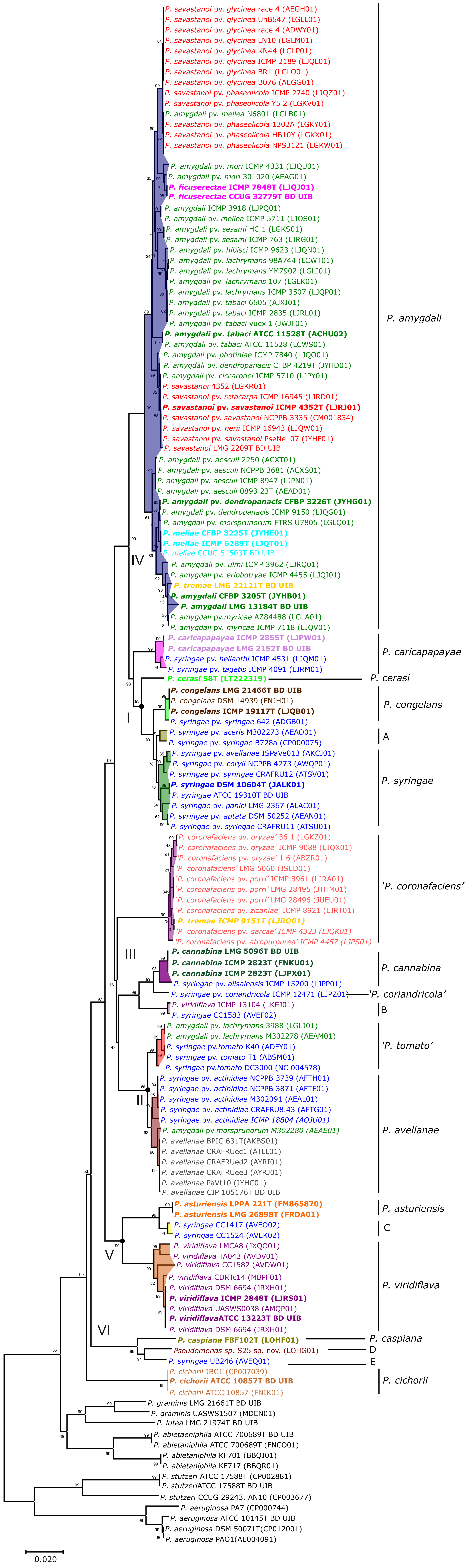


**Supplemental Figure S1.** 3-gene MLSA phylogenetic tree based on the concatenated sequences of 16S rRNA, *gyrB*, and *rpoD* genes of the studied genomes and 15 species type strains of the *P. syringae* phylogenetic group and selected strains as outgroups.
