# Supplementary figures and images for: Clarification of Taxonomic Status within the Pseudomonas syringae Species Group Based on a Phylogenomic Analysis
Source: Front Microbiol. 2017 Dec 7;8:2422. doi: 10.3389/fmicb.2017.02422 (PMC5725466; doi:10.3389/fmicb.2017.02422)

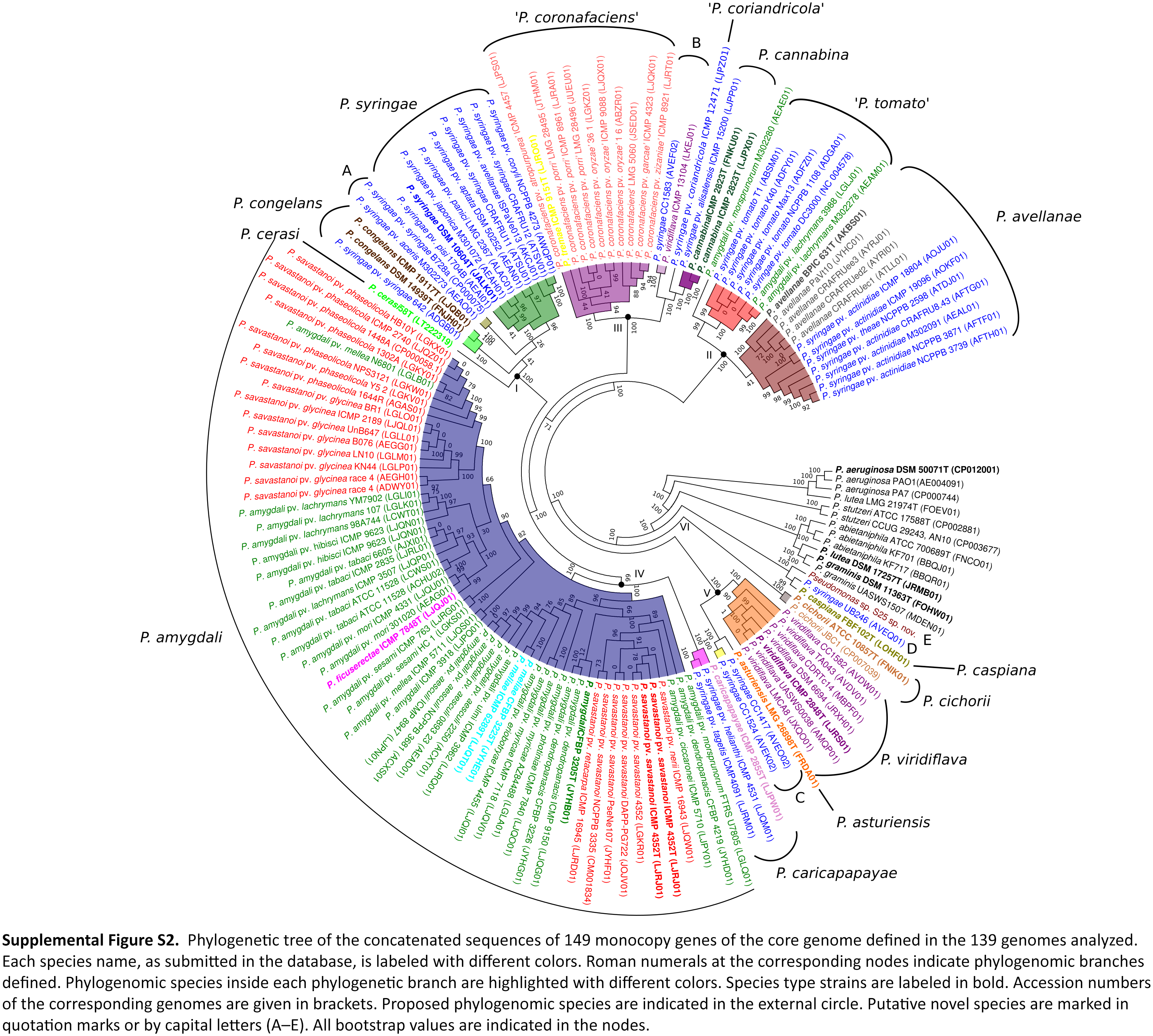

Supplement: Supplementary file 2 [file Image2.TIFF]
