# Supplementary material for: Clarification of Taxonomic Status within the Pseudomonas syringae Species Group Based on a Phylogenomic Analysis
Source: Front Microbiol. 2017 Dec 7;8:2422. doi: 10.3389/fmicb.2017.02422 (PMC5725466; doi:10.3389/fmicb.2017.02422)
Supplement: Supplementary file 3 [file Image3.PDF]

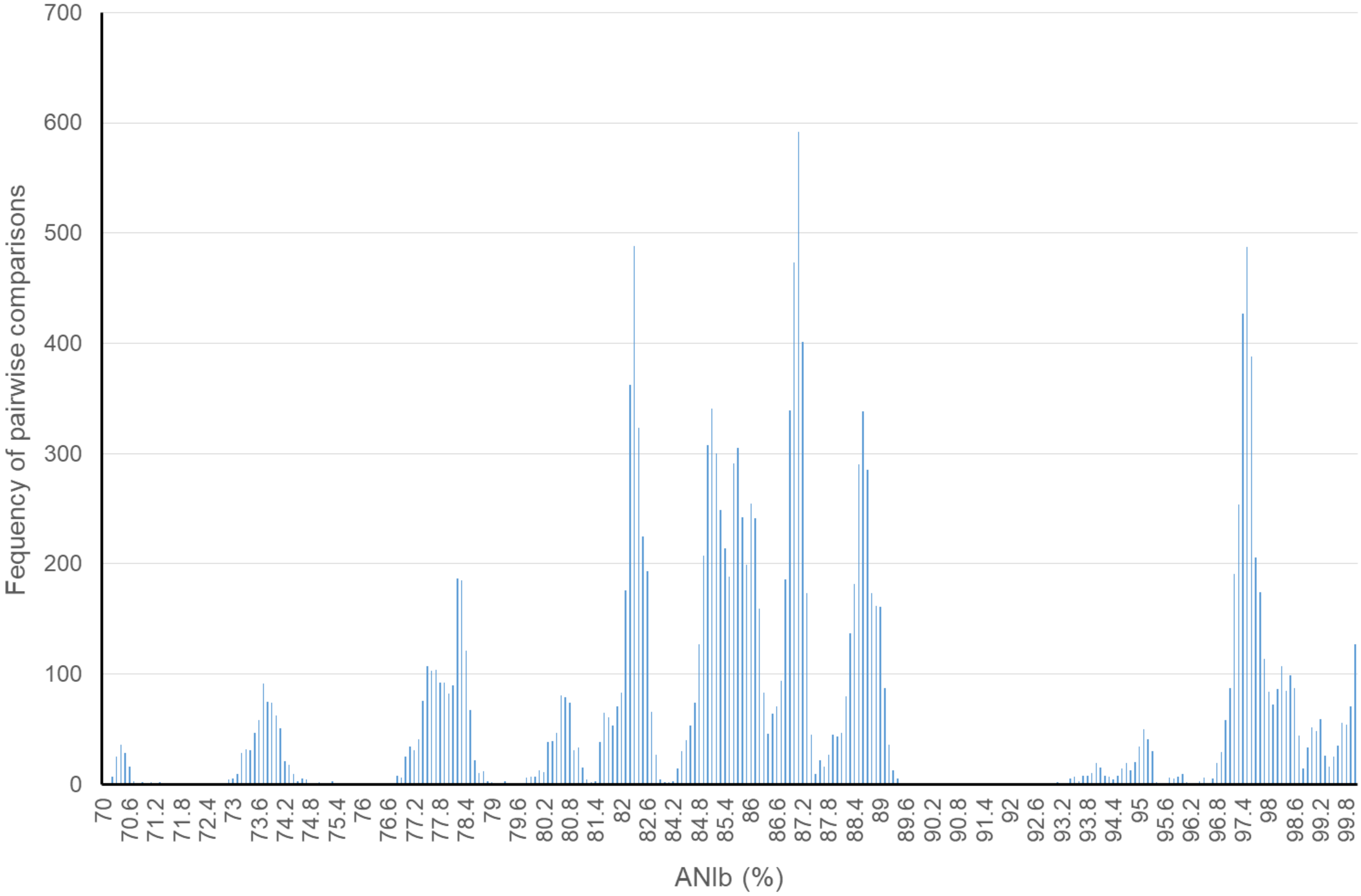

Supplemental Figure S3. ANIb values distribution calculated for all 139 genomes studied. The complete dataset comprised 19,321 reciprocal calculations.
