# Supplementary material for: Clarification of Taxonomic Status within the Pseudomonas syringae Species Group Based on a Phylogenomic Analysis
Source: Front Microbiol. 2017 Dec 7;8:2422. doi: 10.3389/fmicb.2017.02422 (PMC5725466; doi:10.3389/fmicb.2017.02422)
Supplement: Supplementary file 4 [file Image4.PDF]

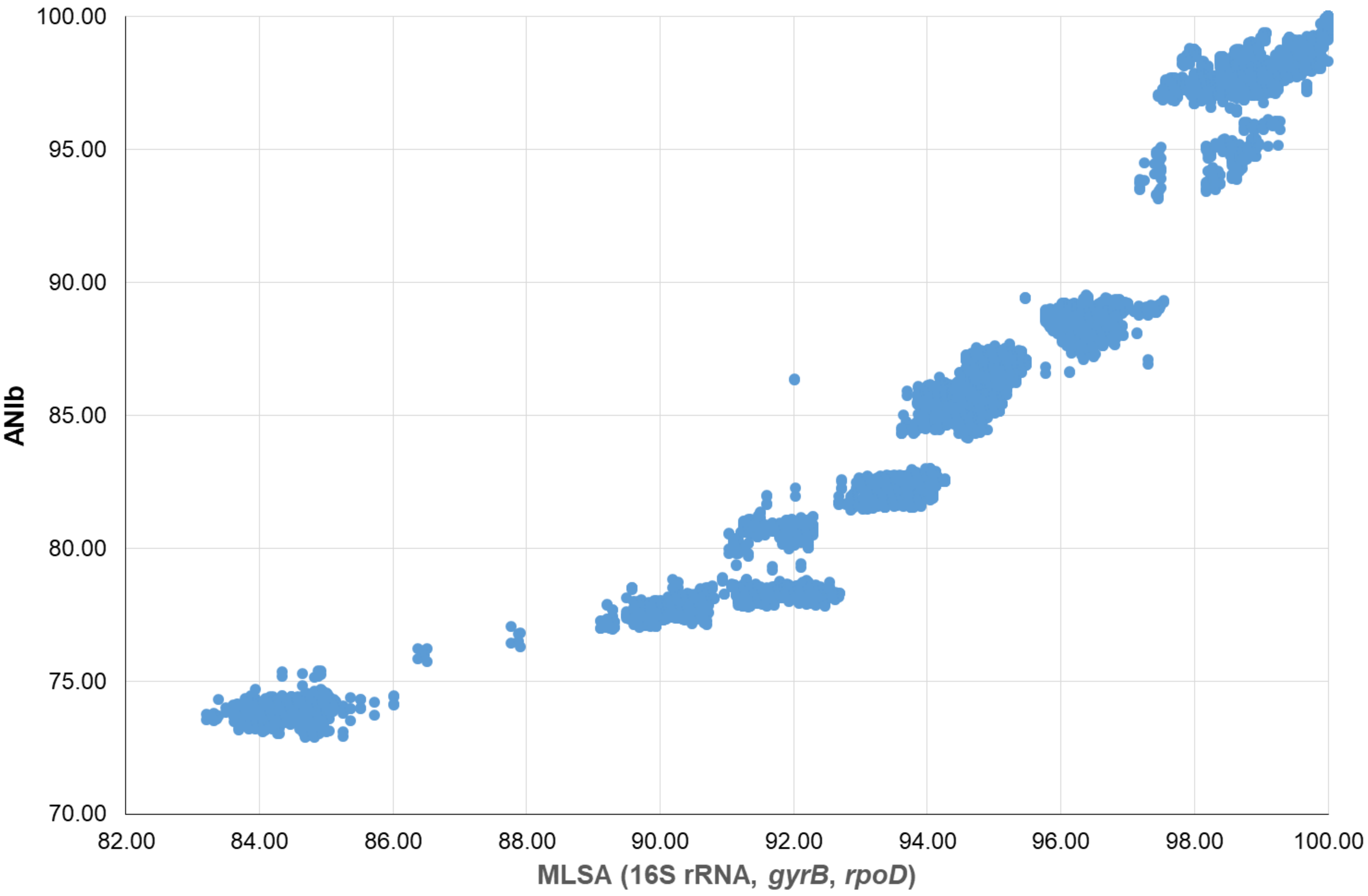

Supplemental Figure S4. Graph representing the relationship between ANIb indices vs. MLSA sequence similarity for the genomes studied. Each dot represents a pairwise comparison.
