# Supplementary material for: Clarification of Taxonomic Status within the Pseudomonas syringae Species Group Based on a Phylogenomic Analysis
Source: Front Microbiol. 2017 Dec 7;8:2422. doi: 10.3389/fmicb.2017.02422 (PMC5725466; doi:10.3389/fmicb.2017.02422)
Supplement: Supplementary file 9 [file Image9.PDF]

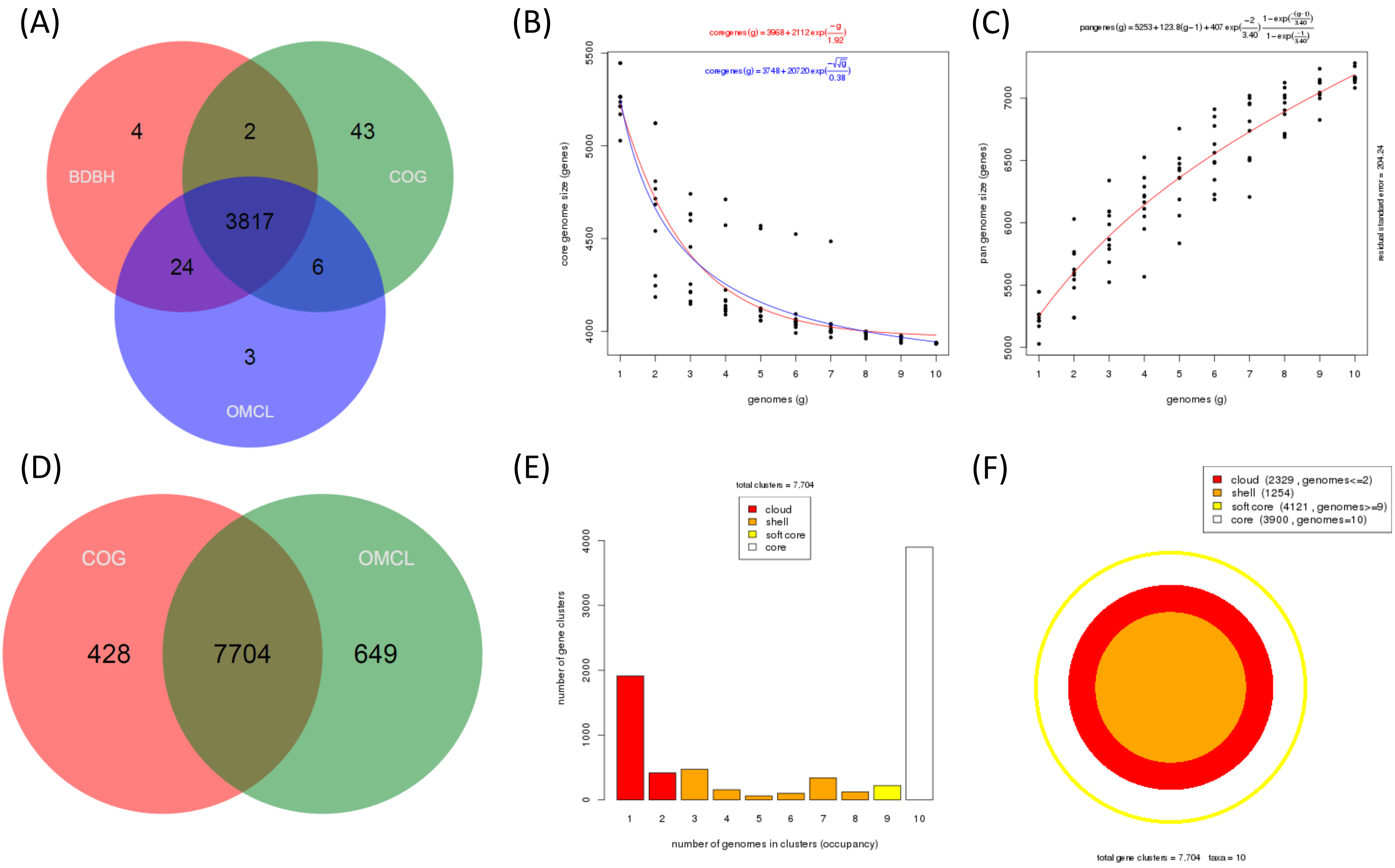

Supplemental Figure S9. Core and pangenome analysis of the 10 strains in the phylogenomic group V (*P. viridifava*, *P. asturiensis* and novel species C). (A) Venn diagram of core genomes generated by the BDBH, COG, and OMCL strategies. (B) Estimate of core genome size with the Tettelin (blue) and Willenbrock (red) fits. (C) Estimate of pangenome size with the Tettelin fit. (D) Venn analysis of pangenomes generated by COG and OMCL. (E and F) Partition of the OMCL pangenomic matrix into shell, cloud, soft-core, and core compartments.

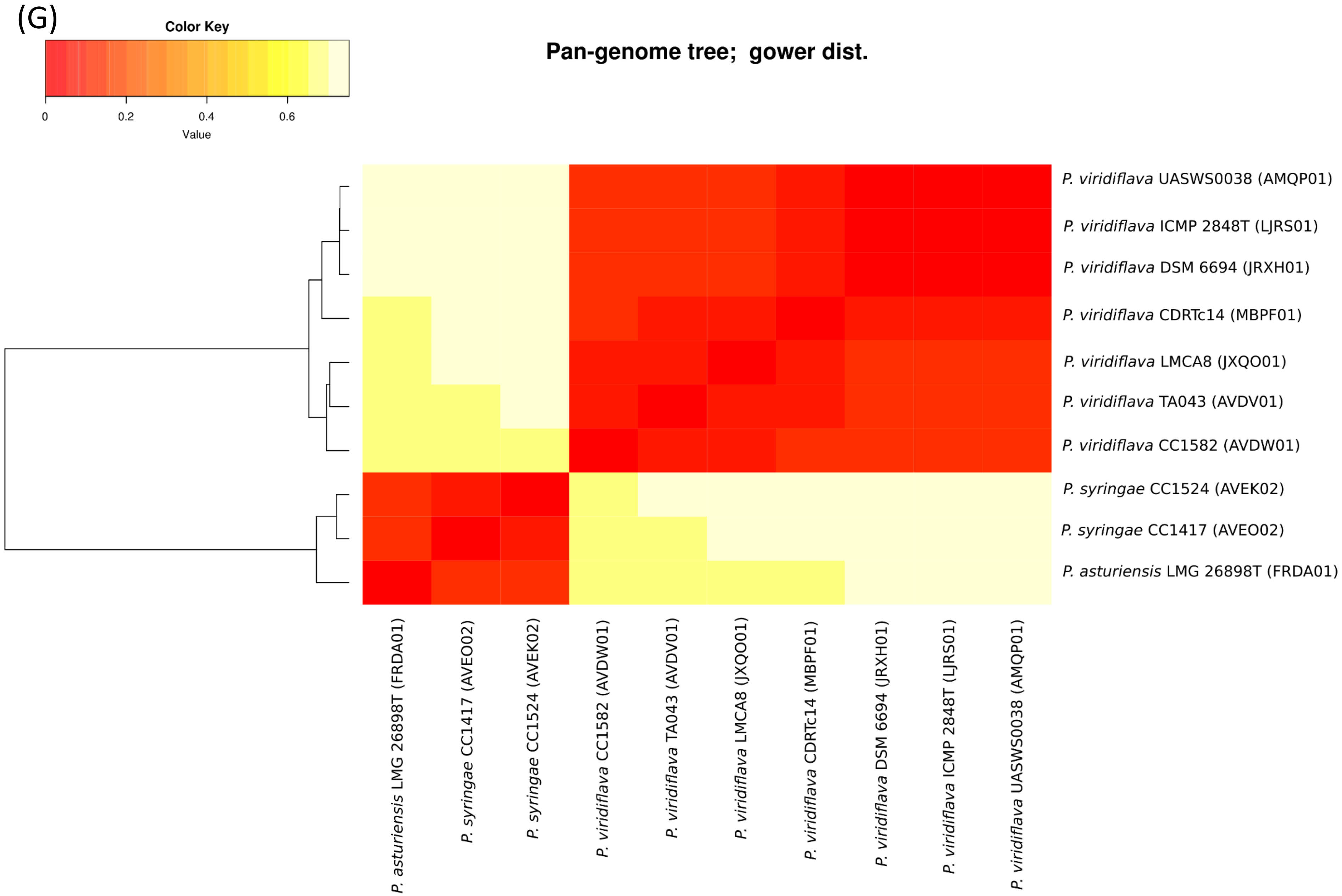

Supplemental Figure S9. Core and pangenome analysis of the 10 strains in the phylogenomic group V (*P. viridifava*, *P. asturiensis* and novel species C). (G) Heatmap representing the degree of similarity of the genomes based on the average amino acid identities of their protein coding genes. High similarities are indicated in red and low similarities in yellow.

(H)

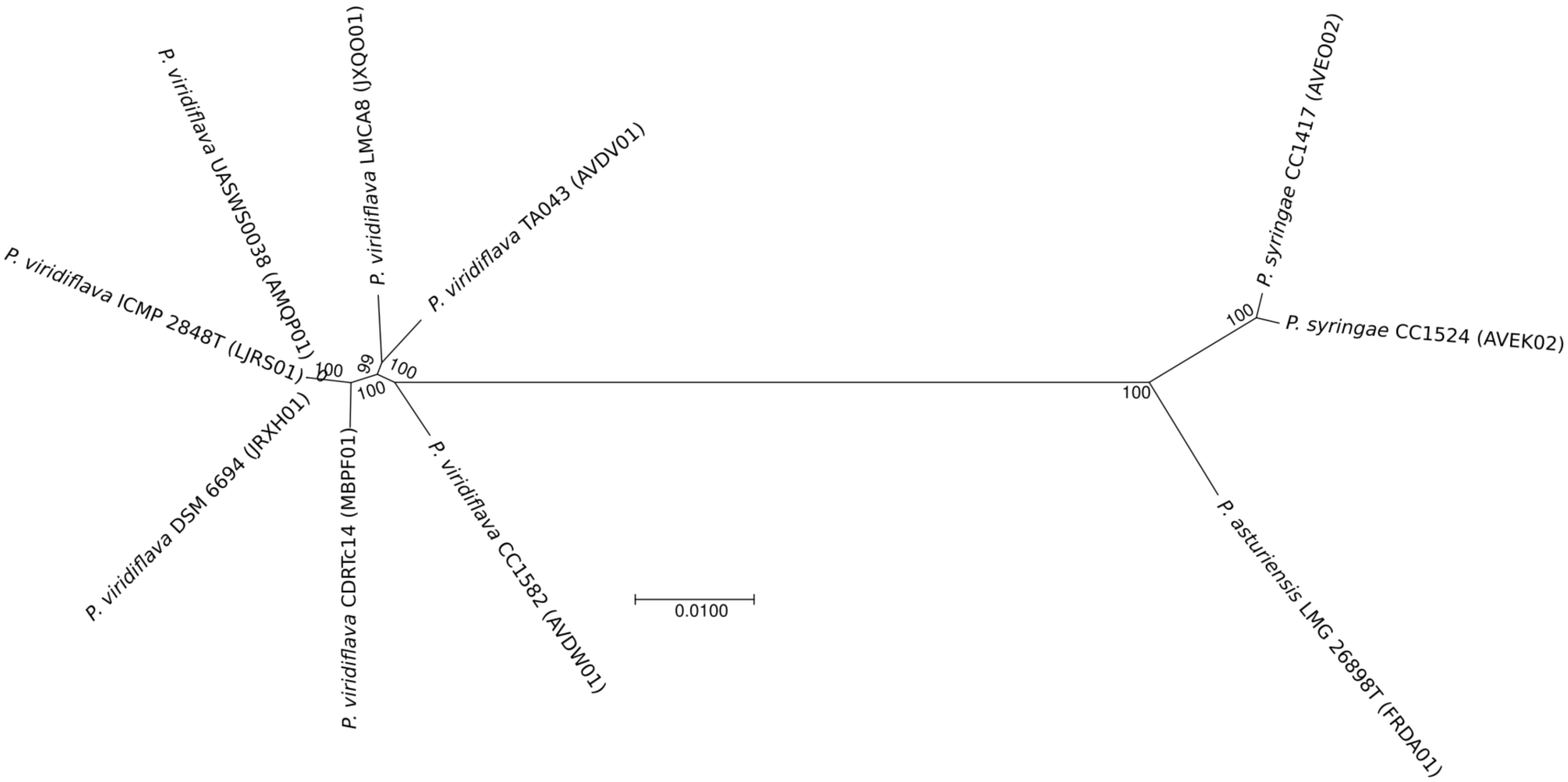

Supplemental Figure S9. Core and pangenome analysis of the 10 strains in the phylogenomic group V (*P. viridifava*, *P. asturiensis* and novel species C). (H) Phylogenetic tree of the concatenated amino acid sequences of 3,817 monocopy proteins of the core genome defined in the 10 genomes analyzed. 1,218,122 amino acid positions were used to construct the tree. Bootstrap values are indicated in the nodes.
