# Supplementary material for: Clarification of Taxonomic Status within the Pseudomonas syringae Species Group Based on a Phylogenomic Analysis
Source: Front Microbiol. 2017 Dec 7;8:2422. doi: 10.3389/fmicb.2017.02422 (PMC5725466; doi:10.3389/fmicb.2017.02422)
Supplement: Supplementary file 11 [file Image11.PDF]

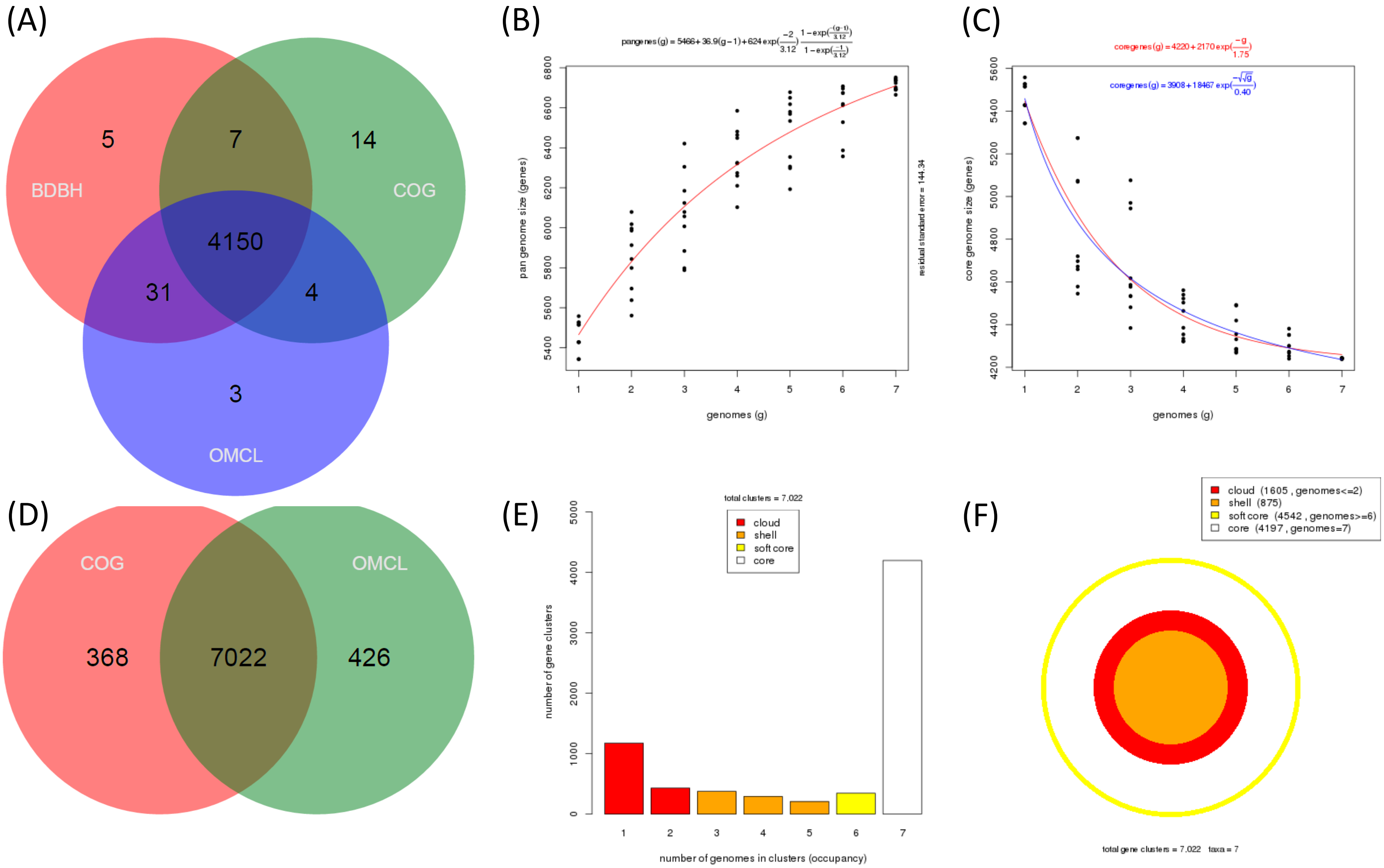

Supplemental Figure S11. Core and pangenome analysis of the 7 strains of '*P. tomato*' phylogenomic species. (A) Venn diagram of core genomes generated by the BDBH, COG, and OMCL strategies. (B) Estimate of core genome size with the Tettelin (blue) and Willenbrock (red) fits. (C) Estimate of pangenome size with the Tettelin fit. (D) Venn analysis of pangenomes generated by COG and OMCL. (E and F) Partition of the OMCL pangenomic matrix into shell, cloud, soft-core, and core compartments.
